# Hospital Re-Organization
*Notes of a lecture given on 14th June, 1942, at a meeting of the Bristol Branch of the Socialist Medical Association.


**Published:** 1942

**Authors:** Somerville Hastings

**Affiliations:** Surgeon-in-charge, Ear, Nose and Throat Department, Middlesex Hospital, London; Chairman of Hospitals and Medical Services Committee, L.C.C.


### HOSPITAL RE-ORGANIZATION.*

BY

Somerville Hastings, M.S., F.R.C.S.,

*Surgeon-in-charge, Ear, Nose and Throat Department,*
*Middlesex Hospital, London;*
*Chairman of Hospitals and Medical Services Committee, L.C.C.*

---

Whilst it is true that many new hospital wards have been built during the war, it is also unfortunately correct that many have been destroyed. It is, of course, difficult to look ahead, but it seems likely that hospitals will be even more necessary after the war than they were before. It may be that people will be poorer; it is certain that there will be a severe housing shortage for many years and, therefore, a greater need for institutional accommodation. Further, treatment, particularly surgical, for conditions that do not cause immediate incapacity, has, in many cases, been postponed during the war. In addition to this, there are war injuries which may be very numerous before victory is won. And lastly, there is the fact that medicine of late years has tended to become more and more mechanized as well as specialized, and this implies an increasing need for hospital accommodation. How is this need to be met after the war?

Most people will, I think, be agreed that it will not be economical or efficient to have two systems of hospital administration—the municipal and the voluntary—doing the same sort of work, and more or less in competition with one another; just as it could never be permissible to have two separate armies with no relation to one another trying to fight a common enemy in the same area.

I should be the last to endeavour to decry the splendid work done in the past, as indeed to-day, by many voluntary hospitals, but the fact remains that the voluntary system has failed to deliver the goods, or in other words to respond to the public need, and because of this municipal hospitals have been developed, and now provide nearly three times as many beds as the voluntary.

Before discussing the development of hospitals in the future, it may be of service to consider what is the ideal system—what, indeed, we must keep before us as an ultimate aim even when we are carrying out the first steps in re-organization. The hospitals of the future

* Notes of a lecture given on 14th June, 1942, at a meeting of the Bristol Branch of the Socialist Medical Association.

will have to be linked up, much more than they are to-day, with all the other preventive and curative agencies. The area of administration, I think, will also have to be large—larger, in fact, than most, if not all, the present Local Authority areas, and should consist probably of not less than a million people. In London, in connection with the municipal hospitals, we have been able to learn from personal experience a good many of the advantages of a large area of administration. Here the seventy-four general and special hospitals are under the charge of a single Medical Officer of Health, and a single Committee for administration, with, of course, the assistance of many sub-committees, both central and local. One great advantage of a large unit is the possibility of bulk purchase of supplies, and here not only is money saved, but quality is maintained, for no firm will be prepared to offend a large customer if this can possibly be avoided. Then there is standardization of equipment and supplies. This, of course, has its dangers, but after careful trial of various types of bedstead, syringe, or other article, at hospitals of different kinds, a standard pattern may be evolved which has many obvious advantages. Then there is the economy of beds because of the possibility of transfer of cases and change of user of any hospital, and much the same applies to medical staff. A few years ago there was a sudden epidemic of influenza in London. The municipal general hospitals were full, the voluntaries could not—or would not—take such cases. Fortunately, however, the incidence of infections disease was low, and within a few days many hundreds of influenza cases were taken into wards especially cleared for them in fever hospitals, and fortunately no cases of cross infection occurred. But perhaps the greatest value to both patients and staff is the possibility of specialization in a large unit. In the London municipal hospitals all plastic cases go to one hospital, nearly all thyroids to another, those needing chest operation to a third, and those requiring radium treatment to one of two. In this way specialized treatment both by doctors and nurses is obtainable, and not only do patients consequently benefit, but the possibilities of valuable research work are greatly increased.

A large hospital area is, therefore, the first essential, and since the consumers, that is the patients, must always come first, and since the final decision on any important point must always be left in their hands, I feel it is necessary that the hospitals in this area should be controlled by a popularly elected Council or Committee. It may, of course, be that a Health Board may first have to be constituted from existing Local Authorities, but eventually the Council in control of this large area should be popularly elected, and should deal, in my opinion, not only with health matters but also with education and most of the other functions now dealt with by Local Government Authorities. I feel sure that all will agree that on such a Board or Council there should be room for co-opted persons, both medical and lay, who have knowledge and experience to put at the service of their fellows. No doubt such a body would divide itself up into various sub-committees, *e.g.* one for domiciliary medical treatment by, I hope, full-time general practitioners and consultants, and another for hospital administration.

Besides dividing up the country in this way into a dozen or more large regions for administration purposes, it will often be convenient for each region to be sub-divided into local units or divisions, the most convenient being one of approximately 100,000 people, and consisting of a small town and the surrounding district. I would not make this a hard and fast rule, and in not a few cases the unit may have to be much larger, but there are many advantages in having such local units in which most of the doctors and other health workers know one another and appreciate each others work. The ideal hospital for such a unit of 100,000 people is one of about a thousand beds, situated, as far as possible, somewhere near the traffic centre of the unit. This hospital should take in all cases that are not dangerous to others, that is to say, everything except tuberculosis, infectious disease and declared mental disease. The rarer specialties, however, might usefully be dealt with each at a single general hospital in the region, as is being done in London. The hospital's services should either be free, or provided by some form of insurance, so that there can be no question of immediate payment by the recipients for these services. The staff should be whole-time, and many of the senior whole-time physicians and surgeons will also see patients in consultation with the general practitioners of the service, either at the Health Centres where the G.P.'s would do much of their work, or in the patient's homes.

It is desirable, I think, that the senior clinical posts in such a hospital should be at least equal in both status and salary with the administrative. Further, I think it is important that every grade, medical and lay, represented in the hospital service, should have, through their appropriate organization, the possibility of direct approach to the Committee of Management for both requests and suggestions.

Lastly, it may be useful to consider how best we can pass by gradual stages from the present hospital arrangements to the unified service which I have tried to sketch.

At the beginning of the war the Government took control of nearly all hospitals under the E.M.S. scheme, and has since paid for beds in these hospitals reserved for casualties and other E.M.S. cases whether occupied or unoccupied. In London, although there were three times as many municipal hospital beds in this service as voluntary, the officers-in-charge of the ten sectors were drawn entirely from the staffs of the voluntary hospitals.

A case could be made for the continuation and extension of the E.M.S. hospital service after the war. It has certainly provided regionalization and some specialization, too, for hospitals for cranial surgery and some other specialities have been developed, but those who have been in close contact with the working of the scheme cannot have failed to have seen the many disadvantages of the divided control in it. There are, however, even more serious disadvantages which make, in my opinion, a continuation of this system after the war impossible. In the first place, it is entirely undemocratic ; it is controlled by the Ministry of Health, and the Ministry of Health Vote comes up on one day per year in Parliament, on which day it is only possible for a very few members to make short speeches and voice grievances. There has been very little planning about the hurriedly conceived E.M.S. scheme. Many Local Authorities which have put much money and energy into the development of their municipal hospitals would greatly resent their being taken over for all time by the Government ; nor, indeed, would it be easy for a hospital system for the whole country to be run from Whitehall. The voluntary hospitals have done exceedingly well from the E.M.S. ; they have received large sums for treatment, or for keeping their beds unoccupied. If the Government were to offer them reasonable payments they would probably refuse, as they have a perfect right to do as private organizations. If the Government were forcibly to take them over, such a course could only be justified if all other private property were similarly nationalized. What should be done, therefore ?

First, I think all hospitals should be inspected and licensed, and except in an emergency only allowed to undertake work for which they have an efficient staff and equipment.

Next, it should be made the statutory duty of the major Local Authorities to provide efficient hospital treatment for all who need it in their area of administration.

In endeavouring to carry out this it would be absurd to ignore the existence of the voluntary hospitals that provided nearly a third of the hospital beds before the war. Clearly, therefore, the Local Authorities must come to terms with these hospitals, and in cases in which there is not sufficient accommodation to provide treatment for all that need it in their own municipal hospitals, they must pay either part or the whole of the cost of treatment of cases that they are compelled to send on to the voluntary hospitals. In return for this the voluntary hospitals must agree that conditions of admission to hospital, as well as of staffing, are similar in all the hospitals of the region, and also that the Local Authorities are given representation on the Boards of Management of the voluntary hospitals proportionate to the amount of money that they provide for their maintenance.

This is, in essence, the scheme which was proposed in Parliament by the Minister of Health on 9th October, 1941. But he went a step further, and very rightly. He said that not only is all this necessary, but that there must also be inspection of hospitals and the formation of a plan so that the resources of each hospital may be estimated and each hospital given its right place in a scheme for the hospital needs of the region. Already the plan for the London area is well advanced and two capable doctors are spending most of their time in working this out. It will then be put before all the hospital authorities concerned, and when the necessary adjustments are made, it will become the plan for London. Strong pressure will be made by the Ministry of Health to induce all hospitals to conform to this plan, and penalties such as the withdrawal of grants for services provided, or refusal to rebuild or extend under a Town Planning Scheme, may be imposed where necessary.

As soon as a scheme of this nature is in good running order it should be possible for the Government to make all hospital treatment provided through the municipality free by Act of Parliament. In that case, anyone requiring treatment and desiring it in any hospital, municipal or voluntary, would be able to obtain it for nothing by application through the municipality. It is difficult to imagine that in these circumstances anyone would apply to the voluntary hospital direct. The voluntary hospitals would thus be filled almost completely by municipal patients, and would thereby pass automatically to the control of the municipal authorities, and for the first time there would be developed a really national hospital system.

